# Screening key lncRNAs for human rectal adenocarcinoma based on lncRNA‐mRNA functional synergistic network

**DOI:** 10.1002/cam4.2236

**Published:** 2019-05-22

**Authors:** Xiongwen Zhu, Dongguo Wang, Qianyuan Lin, Guiyang Wu, Shichao Yuan, Fubo Ye, Qinghao Fan

**Affiliations:** ^1^ Department of Gastrointestinal Surgery Taizhou Municipal Hospital Affiliated with Taizhou University Taizhou China; ^2^ Department of Clinical Lab Medicine Taizhou Municipal Hospital Affiliated with Taizhou University Taizhou China; ^3^ Department of Medical Technology and Pharmacy Renji college of Wenzhou Medical University Wenzhou China

**Keywords:** DElncRNA‐DEmRNA coexpression, DElncRNAs, DEmRNAs, rectal adenocarcinoma, RNA sequencing

## Abstract

**Background:**

Rectal adenocarcinoma (READ) is one of the deadliest malignancies, and the molecular mechanisms underlying the initiation and development of READ remain largely unknown. In this study, we aimed to find key long noncoding RNAs (lncRNAs) and mRNAs in READ by RNA sequencing.

**Methods:**

RNA sequencing was performed to identify differentially expressed mRNAs (DEmRNAs) and lncRNAs (DElncRNAs) between READ and normal tissue. READ‐specific protein‐protein interaction (PPI), DElncRNA‐DEmRNA coexpression, and DElncRNA‐nearby DEmRNA interaction networks were constructed. DEmRNAs and DEmRNAs coexpressed with DElncRNAs were functionally annotated.

**Results:**

A total of 2113 DEmRNAs and 150 DElncRNAs between READ and normal tissue were identified. The PPI network identified several hub proteins, including CDK1, AURKB, CDC6, FOXQ1, NUF2, and TOP2A. The DElncRNA‐DEmRNA coexpression and DElncRNA‐nearby DEmRNA interaction networks identified some hub lncRNAs, including CCAT1, LOC105374879, GAS5, and B3GALT5‐AS1. The colorectal cancer pathway, the intestinal immune network for IgA production and the p53 signaling pathway were three pathways significantly enriched in DEmRNAs and DEmRNAs coexpressed with DElncRNAs. MSH6 coexpressed with two DElncRNAs (LOC105374879 and CASC15) and BCL2 coexpressed with B3GALT5‐AS1 were significantly enriched in the colorectal cancer signaling pathway. TNFRSF17 coexpressed with B3GALT5‐AS1 was enriched in the intestinal immune network for IgA production. CCNB2 coexpressed with LOC105374879 was enriched in the p53 signaling pathway.

**Conclusion:**

A total of four DEmRNAs (MSH6, BCL2, TNFRSF17, and CCNB2) and three DElncRNAs (LOC105374879, CASC15, and B3GALT5‐AS1) may be involved in the pathogenesis of READ; this data may contribute to understanding the mechanisms of READ and the development of therapeutic strategies for READ.

## 1 INTRODUCTION

Colorectal cancer is one of the most common malignant tumors causing cancer‐related deaths and has one of the highest incidence rates among all types of cancer worldwide.^1^ Rectal adenocarcinoma (READ) is a common type of colorectal cancer.^2^ Although advancements in treatments and the prognosis and diagnosis of READ have been achieved through research, its mortality remains high, which may be due to the lack of efficient biomarkers for READ and the unclear mechanisms underlying READ. Hence, identifying efficient biomarkers and deciphering the detailed molecular mechanisms underlying READ are urgently required.

In the field of gene‐gene network analysis, the construction of coexpression networks has opened up enormous possibilities for exploring the role of genes in biological processes.^3^ Coexpression analysis of lncRNAs‐mRNAs is the most commonly used approach to screen potential target genes of lncRNAs and further research on the biological functions of lncRNAs in many kinds of diseases.^4^, ^5^

The advent of high‐throughput genetic analysis means that a large portion of the genome can be transcribed, resulting in the discovery of the extensive transcription of large RNA transcripts named long noncoding RNAs (lncRNAs).^6^, ^7^ Accumulating numbers of reports of aberrant lncRNA expression have demonstrated that lncRNAs may potentially serve as novel independent biomarkers for the early diagnosis and prognosis of and metastasis prediction in various cancer types.^8^, ^9^, ^10^, ^11^ Recently, lncRNA profiling has been performed in several other types of colorectal cancer, which identified novel candidate diagnostic and prognostic biomarkers, such as SNHG6, PVT1, ZFAS1, LINC01555, RP11‐610P16.1, RP11, 108K3.1, and LINC01207.^12^, ^13^ However, research on lncRNA biomarkers in READ is rare.

Owing to the limited research linking lncRNAs with READ, this study aimed to further investigate this issue. In this study, RNA sequencing was performed to identify DEmRNAs and DElncRNAs between READ and normal tissue. READ‐specific protein‐protein interaction (PPI), DElncRNA‐DEmRNA coexpression, and DElncRNA‐nearby DEmRNA interaction networks were constructed. The functional annotation of DEmRNAs and DEmRNAs coexpressed with DElncRNAs was performed. Our study identified potential key genes and lncRNAs in READ and provides further insights into the mechanisms and predictive capacity of lncRNAs in READ.

## 2 MATERIALS AND METHODS

### 2.1 Patients

Three patients with READ were enrolled in our study. Three tissue samples and three paired adjacent normal samples were selected from three cases of READ. The tissue samples were biopsy samples obtained from surgery. The detailed characteristics of the patients are displayed in Table 1. All the participants submitted signed informed consent forms, and the protocols were approved by the ethical committee of our hospital.

**Table 1:** Patient characteristics

	Case 1	Case 2	Case 3
Age (years)	83	82	52
Gender	Male	Female	Male
Diagnostic method	Colonoscopy	Surgery	Colonoscopy
TNM stage	T3N1M0	T4N2M1	T4N2M1
Tumor infiltration	Serosa	Serosa	Serosa
Tumor differentiation	Medium‐grade	Medium low‐grade	Medium low‐grade

### 2.2 RNA isolation, library construction, and sequencing

Total RNA was extracted from the samples using TRIzol reagent (Invitrogen, Carlsbad, CA). A Nanodrop ND‐2000 spectrophotometer (Thermo Scientific, Wilmington, DE) was applied to check the RNA concentration and purity. The integrity of the RNA was detected by agarose gel electrophoresis. The RIN value was obtained by an Agilent 2100 Bioanalyzer. The criteria for cDNA library construction were as follows: (a) total RNA >5 μg; (b) concentration of RNA ≥200 ng/μL; and (3) an OD 260/280 value of 1.8‐2.2.

Ribosomal RNA was removed with a Ribo‐Zero Magnetic kit (EpiCentre, Madison, WI), and the RNA was purified and fragmented into 200‐500‐base pair fragments. The RNA fragments were primed with random hexameric primers, and the first cDNA strand was synthesized, with the second cDNA strand synthesized with dUTP instead of dTTP. After purification with AMPure XP Beads (Beckman Coulter, Brea, CA), end repair, adenylation of the 3′ ends and adapter ligation were performed. Polymerase chain reaction (PCR) was performed to construct a library for the high‐throughput sequencing of lncRNA, and the mRNA from the second cDNA strand was digested using UNG enzyme (Illumina, Inc, San Diego, CA). All libraries used for the high‐throughput sequencing of lncRNAs and mRNAs were amplified by 15 cycles of PCR. The quality of the library was assessed using the Agilent 2100 Bioanalyzer and ABI StepOnePlus Real‐Time PCR System. The sequencing of lncRNAs and mRNAs was performed on an Illumina HiSeq Xten platform (Illumina, San Diego, CA).

### 2.3 Quality control of raw sequences and mapping of clean reads

FASTQ sequence data were obtained from the RNA‐seq data using Base Calling V 0.11.4 (http://www.bioinformatics.babraham.ac.uk/projects/fastqc/). Low‐quality reads, including adaptor sequences, sequences with a quality score <20, and sequences with an N base percentage of the raw reads >10% were removed using Cutadapt V 1.9.1 (https://cutadapt.readthedocs.io/en/stable/) with TopHat (http://tophat.cbcb.umd.edu/) and Ensembl gene annotation. The clean reads were aligned with the human reference genome, Ensembl GRCh38.p7 (ftp://ftp.ncbi.nlm.nih.gov/genomes/Homo_sapiens). The expression of mRNAs and lncRNAs was determined using Cuffquant V 2.2.1.

### 2.4 Differential expression analysis of mRNAs and lncRNAs

The mRNAs and lncRNAs were quantified using Cuffquant V 2.2.1. Cuffdiff (http://cufflinks.cbcb.umd.edu/) uses the quantitative results of Cuffquant to compare differences in the expression of each mRNA and lncRNA in READ and normal tissue. mRNAs and lncRNAs with a *P*‐value <0.05 and |log_2_ fold change |>1 were significantly differentially expressed mRNAs (DEmRNAs) and differentially expressed lncRNAs (DElncRNAs), respectively. A heat map of the DEmRNAs and DElncRNAs in READ was obtained by heatmap.2 (http://127.0.0.1:28428/library/gplots/html/heatmap.2.html).

### 2.5 Functional annotation

GeneCodis 3 (http://genecodis.cnb.csic.es/analysis) is an online software tool for functional annotation analysis used to reveal the biological functions related to large lists of genes. Gene Ontology (GO) classification (biological process, cellular component, and molecular function) is a major bioinformatics analysis method for annotating genes. The Kyoto Encyclopedia of Genes and Genomes (KEGG) is a database used to determine the biological systems associated with the output of high‐throughput experimental technologies. GO classification and KEGG pathway enrichment analyses were performed using GeneCodis 3. An false discovery rate (FDR) <0.05 was used to indicate statistical significance.

### 2.6 PPI network construction

The top 100 upregulated or downregulated DEmRNAs in READ were used to build a PPI network using Biological General Repository for Interaction Datasets (BioGRID) (http://thebiogrid.org/) and Cytoscape 3.5.0 (http://www.cytoscape.org/). We used nodes to represent proteins and edges to represent the interactions between two proteins.

### 2.7 DEmRNA‐DElncRNA interaction analysis

To identify DEmRNAs near DElncRNAs with cis‐regulatory effects, DEmRNAs transcribed within a 100 kb window up‐ or downstream of DElncRNAs in READ and normal controls were identified. In addition, DEmRNAs coexpressed with DElncRNAs were identified. Pairwise Pearson correlation coefficients between DEmRNAs and DElncRNAs were calculated. DElncRNA‐DEmRNA pairs with *P* < 0.001 and | r | ≥0.98 were defined as significant mRNA‐lncRNA coexpression pairs.

## 3 RESULTS

### 3.1 DEmRNAs and DElncRNAs in READ

**Figure 1:**
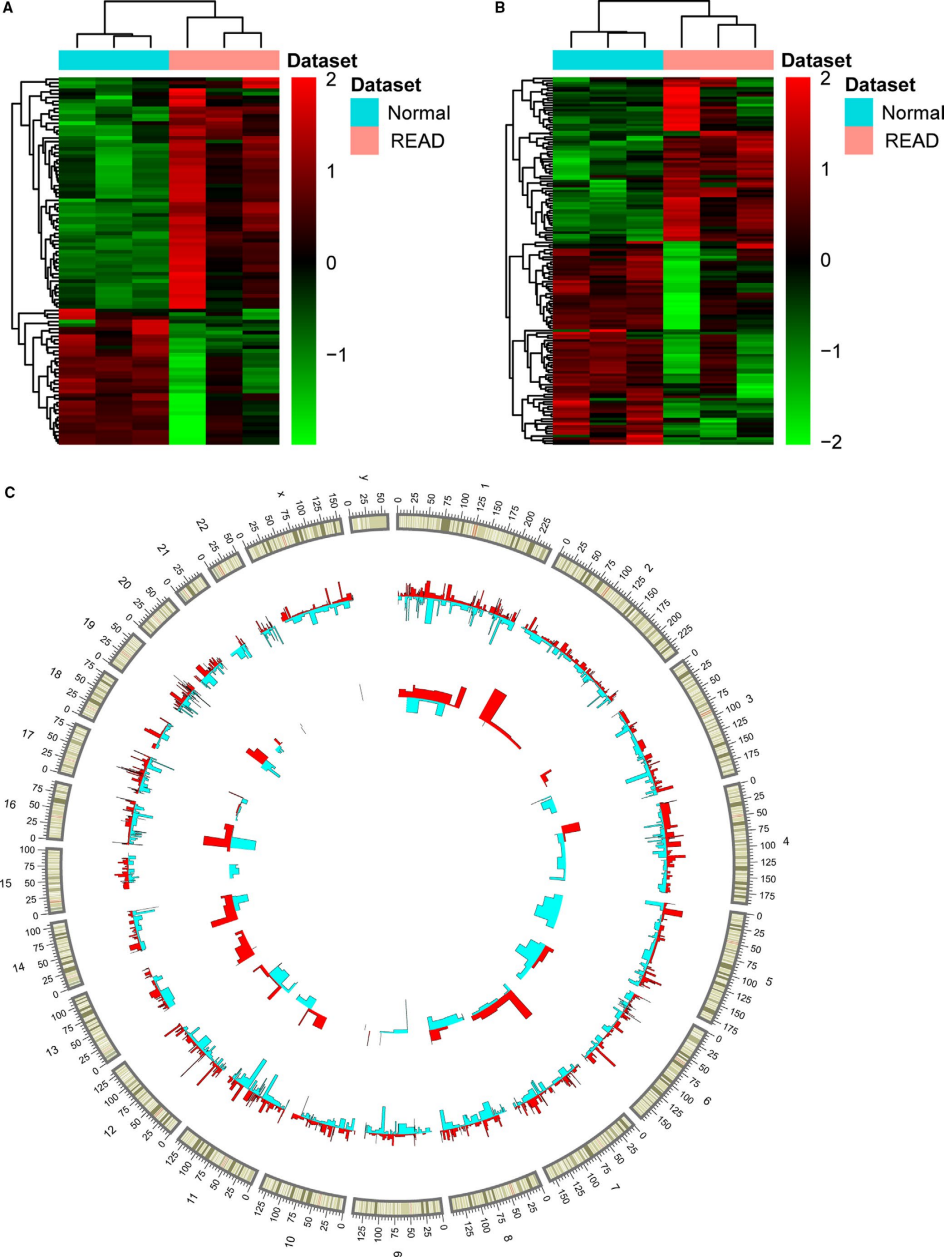
Heat map of the top 100 DEmRNAs and all of DElncRNAs between READ and normal tissues. (A) DEmRNAs. (B) DElncRNAs. Rows and columns represent DElncRNAs/DEmRNAs and tissue samples, respectively. The color scale indicates expression levels. (C) Circos plots representing the distribution of DElncRNAs and DEmRNAs on chromosomes. The outer layer cycle is the chromosome map of the human genome. The inner layers represent the distribution of DEmRNAs and DElncRNAs on different chromosomes, respectively. Red and blue colors represent up‐ and downregulation, respectively

The raw data has been uploaded to Gene Expression Omnibus (GEO) (GSE128969, https://www.ncbi.nlm.nih.gov/geo/query/acc.cgi?acc=GSE128969). A total of 2113 DEmRNAs (809 downregulated and 1304 upregulated mRNAs) and 150 DElncRNAs (81 downregulated and 69 upregulated lncRNAs) between READ and normal tissue were identified with an FDR < 0.05 and a |Log_2_fold change|>1. The top 20 most significant DEmRNAs and DElncRNAs are displayed in Tables 2 and 3, respectively. Heatmaps of the top 100 DEmRNAs and all of DElncRNAs between READ and normal tissue are shown in Figure 1A,B, respectively. Circos plots representing the distribution of DElncRNAs and DEmRNAs on chromosomes are shown in Figure 1C.

**Table 2:** The top 20 DEmRNAs andin READ

ID	Symbol	log_2_FC	*P*‐value	FDR	Up/down
3854	KRT6B	7.73832	5.00E‐05	0.002611	Up
342667	STAC2	6.74383	5.00E‐05	0.002611	Up
28234	SLCO1B3	6.69561	5.00E‐05	0.002611	Up
5655	KLK10	5.91643	5.00E‐05	0.002611	Up
221416	C6orf223	5.08019	5.00E‐05	0.002611	Up
90161	HS6ST2	5.06794	5.00E‐05	0.002611	Up
1800	DPEP1	5.02634	5.00E‐05	0.002611	Up
1767	DNAH5	4.95572	5.00E‐05	0.002611	Up
990	CDC6	4.9442	5.00E‐05	0.002611	Up
9271	PIWIL1	4.94295	5.00E‐05	0.002611	Up
55532	SLC30A10	−4.3053	5.00E‐05	0.002611	Down
229	ALDOB	−4.02838	5.00E‐05	0.002611	Down
2346	FOLH1	−4.01874	5.00E‐05	0.002611	Down
374569	ASPG	−3.97546	5.00E‐05	0.002611	Down
10022	INSL5	−3.84557	5.00E‐05	0.002611	Down
5320	PLA2G2A	−3.76694	5.00E‐05	0.002611	Down
6689	SPIB	−3.67292	5.00E‐05	0.002611	Down
8115	TCL1A	−3.46401	5.00E‐05	0.002611	Down
1380	CR2	−3.36749	5.00E‐05	0.002611	Down
266675	BEST4	−3.34166	5.00E‐05	0.002611	Down

**Table 3:** The top 20 DElncRNAs. in READ

ID	Symbol	log_2_FC	*P*‐value	FDR	Up/down
503638	LINC01296	5.37396	5.00E‐05	0.002611	Up
652995	UCA1	4.39663	5.00E‐05	0.002611	Up
105369370	LOC105369370	4.23597	5.00E‐05	0.002611	Up
102723961	LOC102723961	3.53756	5.00E‐05	0.002611	Up
100507056	CCAT1	3.34382	5.00E‐05	0.002611	Up
105374879	LOC105374879	2.59434	5.00E‐05	0.002611	Up
407975	MIR17HG	2.18303	5.00E‐05	0.002611	Up
105370108	LOC105370108	4.57119	0.0001	0.004682	Up
105376380	LOC105376380	3.45882	0.0002	0.007904	Up
60674	GAS5	1.20787	0.0002	0.007904	Up
105377567	LOC105377567	−2.77552	0.0001	0.004682	Down
283422	LINC01559	−1.28123	0.00015	0.006442	Down
283663	LINC00926	−2.10879	0.00035	0.011778	Down
114041	B3GALT5‐AS1	−1.89195	0.0004	0.012989	Down
284185	LINC00482	−1.90215	0.00055	0.016205	Down
–	LOC101926893	−3.61245	0.0006	0.017319	Down
–	LOC100507616	−2.896	0.0007	0.019391	Down
149837	LINC00654	−1.49029	0.0007	0.019391	Down
100289019	SLC25A25‐AS1	−1.13489	0.0008	0.021382	Down
–	LOC389332	−1.9268	0.00095	0.023864	Down

### 3.2 Functional annotation of DEmRNAs in READ

**Figure 2:**
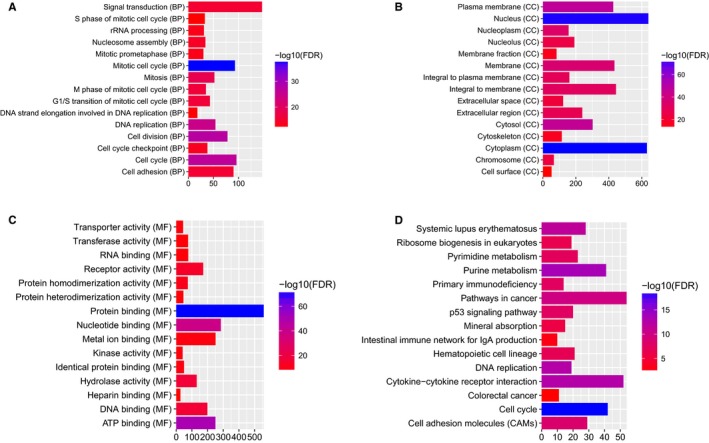
The top 15 significantly enriched GO terms and KEGG pathways for DEmRNAs in READ. The *x*‐axis shows ‐log FDR, and the *y*‐axis shows GO terms or KEGG pathways. (A) Biological process. (B) Cellular component. (C) Molecular function. (D) KEGG pathways

DEmRNAs were used for GO and KEGG enrichment analyses. GO enrichment analysis showed that the DEmRNAs were significantly enriched in the mitotic cell cycle (FDR = 3.62E‐38), cell division (FDR = 4.10E‐28), cytoplasm (FDR = 1.70E‐75), nucleus (FDR = 5.13E‐ 75), protein binding (FDR = 2.82E‐72), and ATP binding (FDR = 4.42E‐51) terms. The top 15 GO terms for the DEmRNAs in READ are displayed in Figure 2A‐C. KEGG pathway enrichment analysis revealed that the p53 signaling pathway (FDR = 2.05E‐08), intestinal immune network for IgA production (FDR = 9.91E‐04), and colorectal cancer (FDR = 3.49E‐03) pathway were three significantly enriched pathways in READ. The top 15 most significantly enriched KEGG pathways for the DEmRNAs in READ are shown in Figure 2D.

### 3.3 READ‐specific PPI network construction

**Figure 3:**
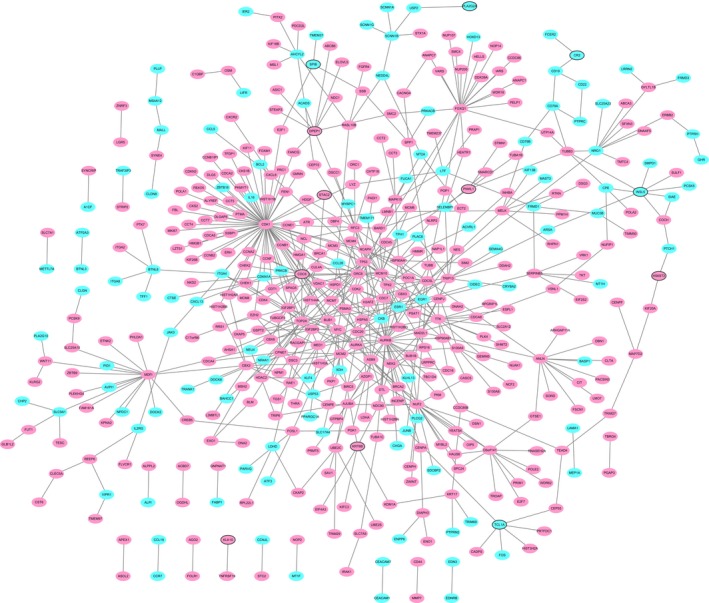
READ‐specific PPI network. Ellipses are used to represent nodes, and lines are used to represent edges. Red and blue represent up‐ and downward adjustments, respectively. The black border indicates the top 10 up‐ and downregulated proteins

A PPI network of the top 100 up‐ and downregulated DEmRNAs consisted of 464 nodes and 591 edges (Figure 3). CDK1 (degree = 67), AURKB (degree = 34), CDC6 (degree = 20), FOXQ1 (degree = 20), NUF2 (degree = 19), and TOP2A (degree = 18) were considered hub proteins.

### 3.4 DElncRNA‐DEmRNA coexpression network

**Figure 4:**
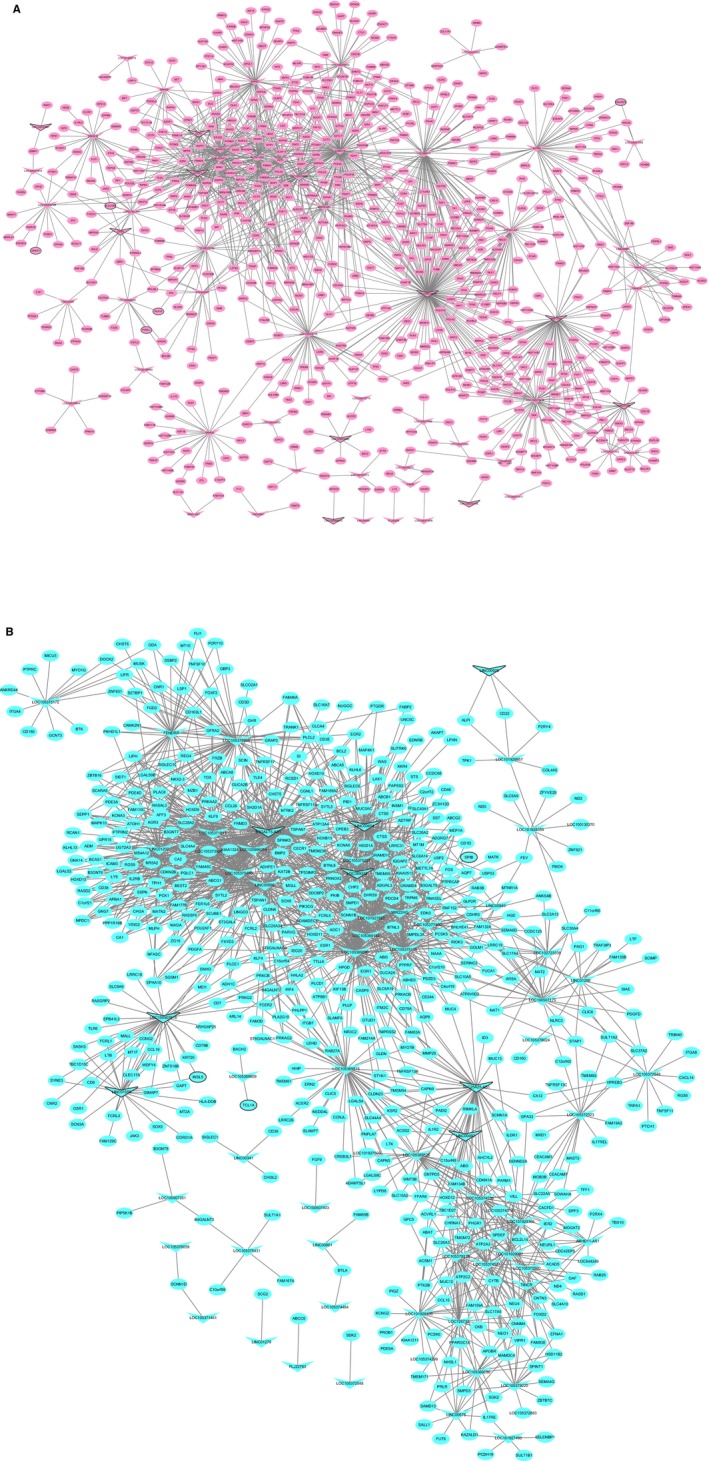
Positively coexpressed DElncRNA‐DEmRNA network. Ellipses and inverted triangles represent DEmRNAs and DElncRNAs, respectively. Red and blue colors represent up‐ and downregulation, respectively. The black border indicates the top 10 up‐ and downregulated DElncRNAs and DEmRNAs

A total of 5122 DElncRNA‐DEmRNA coexpression pairs including 150 DElncRNAs and 2110 DEmRNAs were identified with an absolute value of the Pearson correlation coefficient | *r* | ≥ 0.98 and a *P*‐value <0.001. We obtained a total of 3293 lncRNA‐mRNA pairs that were positively coexpressed and 1829 lncRNA‐mRNA pairs that were negatively coexpressed. The positively coexpressed DElncRNA‐DEmRNA network (Figure 4) consisted of 1364 nodes and 3293 edges, and its hub lncRNAs were CCAT1 (degree = 87), LOC105374879 (degree = 164), MIR17HG (degree = 72), UCA1 (degree = 35), and B3GALT5‐AS1 (degree = 141).

**Figure 5:**
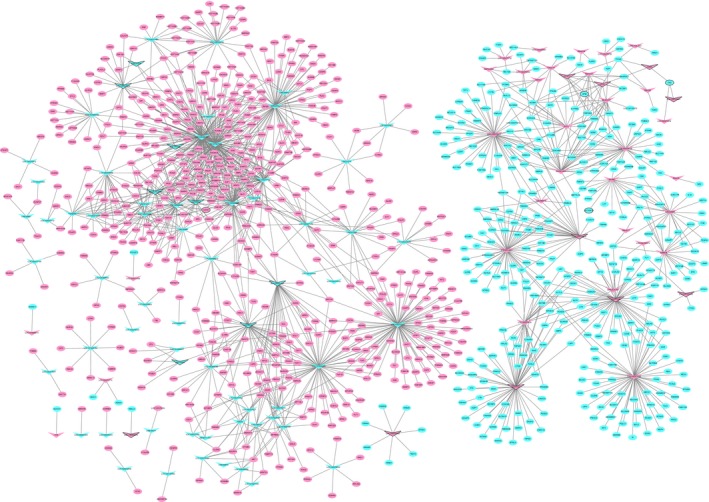
Negatively coexpressed DElncRNA‐DEmRNA network. Ellipses and inverted triangles represent DEmRNAs and DElncRNAs, respectively. Red and blue colors represent up‐ and downregulation, respectively. The black border indicates the top 10 up‐ and downregulated DElncRNAs and DEmRNAs

The negatively coexpressed DElncRNA‐DEmRNA network (Figure 5) consisted of 1049 nodes and 1829 edges, and its hub lncRNAs were LOC105374879 (degree = 33), LINC00482 (degree = 42), B3GALT5‐AS1 (degree = 31), and MIR17HG (degree = 55).

### 3.5 Functional annotation of DEmRNAs coexpressed with DElncRNAs

**Figure 6:**
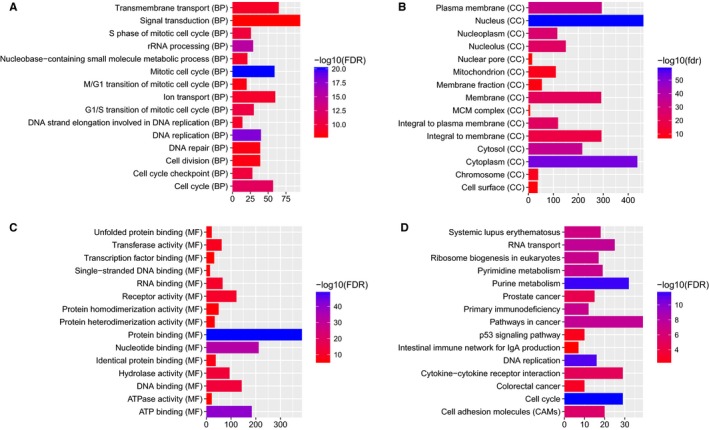
Top 15 significantly enriched GO terms and KEGG pathways for DEmRNAs coexpressed with DElncRNAs in READ. The x‐axis shows ‐log FDR, and the y‐axis shows GO terms or KEGG pathways. (A) Biological process. (B) Cellular component. (C) Molecular function. (D) KEGG pathways

**Figure 7:**
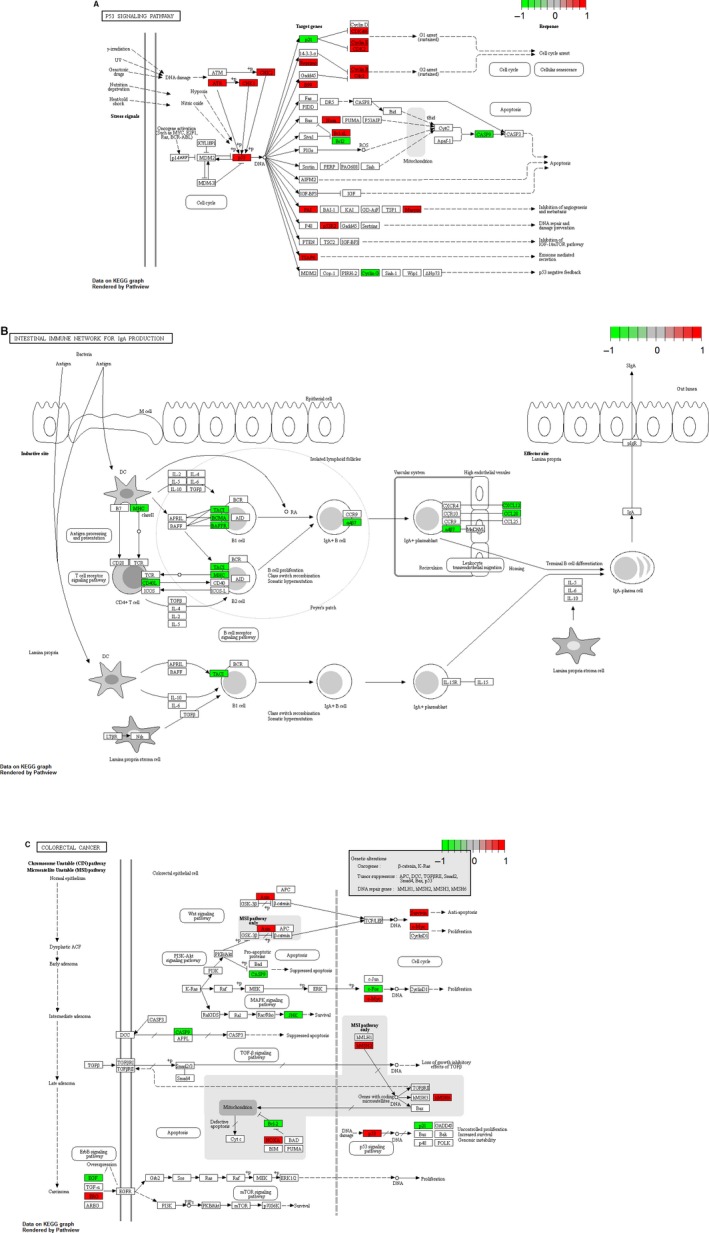
READ pathways (p53 signaling pathway, intestinal immune network for IgA production, and colorectal cancer pathway) enriched in DEmRNAs during READ. The red and green rectangles represent components regulated by DEmRNAs that are enriched in READ

According to the GO enrichment analysis of DEmRNAs with an FDR < 0.05, the mitotic cell cycle (FDR = 8.66E‐21), DNA replication (FDR = 8.36E‐19), nucleus (FDR = 5.30E‐60), cytoplasm (FDR = 6.70E‐53), protein binding (FDR = 2.47E‐50), and ATP binding (FDR = 5.58E‐41) terms were the most significantly enriched GO terms. The top 15 GO terms of the DEmRNAs in READ are displayed in Figure 6A‐C. After KEGG pathway enrichment analysis (FDR < 0.05), we found that the cell cycle (FDR = 1.36E‐12), purine metabolism (FDR = 2.74E‐12), and DNA replication (FDR = 5.16E‐12) pathways were the three most significantly enriched pathways in READ. The top 15 most significantly enriched KEGG pathways for DEmRNAs in READ are shown in Figure 6D. The p53 signaling pathway (FDR = 0.0023), intestinal immune network for IgA production (FDR = 0.0084), and colorectal cancer pathway (FDR = 0.0014) were three READ‐related pathways. The p53 signaling pathway, intestinal immune network for IgA production and colorectal cancer pathway are displayed in Figure 7.

### 3.6 DElncRNA‐nearby DEmRNA interaction network

**Figure 8:**
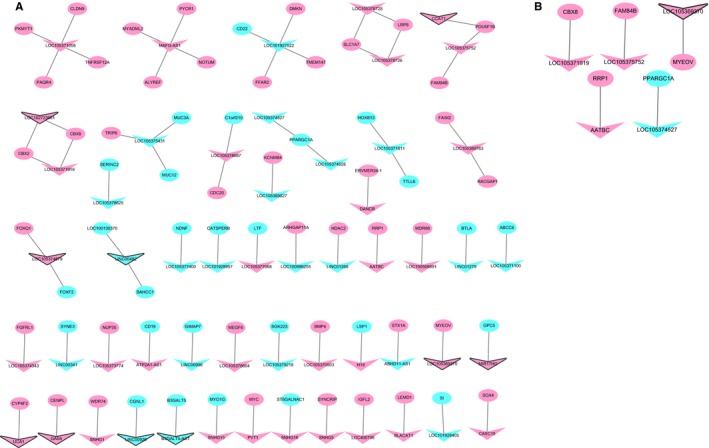
DElncRNA‐nearby DEmRNA interaction network in READ. (A) DElncRNA‐nearby DEmRNA interaction network. (B) Interaction network showing the overlap of the DElncRNA‐DEmRNA coexpression network with the DElncRNA‐nearby DEmRNA interaction network. Ellipses and inverted triangles represent DEmRNAs and DElncRNAs, respectively. Red and blue colors represent up‐ and downregulation, respectively. The black border indicates the top 10 up‐ and downregulated

The functions of most lncRNAs remain unknown. We hypothesized that lncRNAs may exert their functions by regulating nearby genes. A total of 75 DElncRNA‐nearby target DEmRNA pairs were obtained that consisted of 54 DElncRNAs and 69 DEmRNAs (Figure 8A). Ten DElncRNAs with the closest DEmRNAs were CCAT1, LOC102723961, LOC105369370, LOC105374879, MIR17HG, UCA1, GAS5, LINC00926, B3GALT5‐AS1, and LINC00482, which were nearby 1, 2, 1, 2, 1, 1, 1, 1, 1, and 2 DEmRNAs, respectively. The DElncRNA‐nearby DEmRNA pairs in which the DEmRNA was coexpressed with the DElncRNA are displayed in Table 4. After looking for overlaps in the DElncRNA‐DEmRNA coexpression network and the DElncRNAs‐nearby DEmRNAs interaction network, we obtained a total of five lncRNA‐mRNA pairs including five DElncRNAs and five DEmRNAs (Figure 8B). Among these, LOC105369370 was within the top 10 DElncRNAs. Moreover, MYEOV was not only an DEmRNA nearby LOC105369370 but was also coexpressed with LOC105369370.

**Table 4:** The DElncRNAs‐nearby DEmRNAs pairs

Chr	lncRNA			mRNA		
	Symbol	Start − 100kb	End + 100kb	Symbol	Start	End
chr8	CCAT1	127107381	127319268	POU5F1B	127244636	127482139
chr17	LOC102723961	79715942	79923284	CBX2	79776253	79787650
chr17	LOC102723961	79715942	79923284	CBX8	79794376	79797116
chr11	LOC105369370	69266824	69472512	MYEOV	69294137	69297287
chr6	LOC105374879	1184930	1391486	FOXQ1	1312439	1314758
chr6	LOC105374879	1184930	1391486	FOXF2	1389833	1395597
chr13	MIR17HG	91247819	91454575	GPC5	91398618	92867237
chr19	UCA1	15727044	15936321	CYP4F2	15878023	15898120
chr1	GAS5	173763247	173967987	CENPL	173799549	173824639
chr11	SNHG1	62751987	62955888	WDR74	62832233	62841809
chr15	LINC00926	57200364	57407769	CGNL1	57376486	57550727
chr21	B3GALT5‐AS1	39497146	39712822	B3GALT5	39612939	39662889
chr17	LINC00482	81202823	81409248	LOC100130370	81375496	81392947
chr17	LINC00482	81202823	81409248	BAHCC1	81395430	81466332
chr7	SNHG15	44883027	45086660	MYO1G	44962660	44979105
chr8	LOC105375752	127040057	127269518	POU5F1B	127244636	127482139
chr8	LOC105375752	127040057	127269518	FAM84B	126552437	127049451
chr8	PVT1	127694532	128201253	MYC	127736068	127741434
chr17	SNHG16	76457763	76665348	ST6GALNAC1	76617768	76643838
chr7	LOC105375431	100842058	101069565	TRIP6	100867327	100873454
chr7	LOC105375431	100842058	101069565	MUC12	100969622	101018949
chr7	LOC105375431	100842058	101069565	MUC3A	100942058	100969565
chr6	SNHG5	85577006	85778733	SYNCRIP	85607783	85643862
chr19	LOC400706	45957677	46177629	IGFL2	46078512	46203062
chr1	BLACAT1	205273251	205556086	LEMD1	205373251	205456086
chr6	CASC15	21566443	22294400	SOX4	21593740	21598619
chr4	DANCR	52612149	52823436	ERVMER34‐1	52743516	52753572
chr17	MAFG‐AS1	81827828	82030753	ALYREF	81887834	81900533
chr17	MAFG‐AS1	81827828	82030753	MYADML2	81939644	81947233
chr17	MAFG‐AS1	81827828	82030753	PYCR1	81932383	81937328
chr17	MAFG‐AS1	81827828	82030753	NOTUM	81952506	81961181
chr12	LOC105369827	70368087	70616501	KCNMB4	70366219	70434292
chr19	LOC101927522	35305606	35534730	FFAR2	35447964	35451767
chr19	LOC101927522	35305606	35534730	CD22	35329165	35347361
chr19	LOC101927522	35305606	35534730	DMKN	35497216	35513678
chr19	LOC101927522	35305606	35534730	TMEM147	35533337	35547527
chr1	LOC105378625	31348257	31549586	SERINC2	31409564	31434680
chr16	LOC105371100	16049564	16323616	ABCC6	16149564	16223616
chr3	LOC101928405	165050005	165258164	SI	164978897	165083824
chr17	LOC105371919	79723794	79927704	CBX2	79776253	79787650
chr17	LOC105371919	79723794	79927704	CBX8	79794376	79797116
chr1	LOC105378687	43254683	43458673	C1orf210	43281864	43285840
chr1	LOC105378687	43254683	43458673	CDC20	43358954	43363203
chr4	LOC105377400	120972758	121181901	NDNF	121035626	121072518
chr14	LOC101928957	91420514	91677823	CATSPERB	91580773	91732086
chr3	LOC105377068	46314778	46524092	LTF	46436004	46485234
chr4	LOC105374527	23669093	23883980	PPARGC1A	23792020	24472975
chr15	LOC100996255	32436758	32680609	ARHGAP11A	32615143	32639949
chr1	LOC105378728	53126891	53428149	SLC1A7	53087178	53142632
chr1	LOC105378728	53126891	53428149	LRP8	53226891	53328149
chr6	LINC01268	113768012	113980812	HDAC2	113936155	114342388
chr21	AATBC	43705757	43912567	RRP1	43789536	43804102
chr12	LOC100506691	121963289	122168560	WDR66	121918556	122003927
chr17	LOC105371811	48632983	48843465	HOXB13	48724762	48728749
chr17	LOC105371811	48632983	48843465	TTLL6	48762230	48817253
chr16	LOC105371058	2977271	3187993	PAQR4	2969244	2980539
chr16	LOC105371058	2977271	3187993	PKMYT1	2969244	2980539
chr16	LOC105371058	2977271	3187993	CLDN9	3012455	3014505
chr16	LOC105371058	2977271	3187993	TNFRSF12A	3020311	3022383
chr3	LINC01279	112496793	112701969	BTLA	112458789	112499756
chr4	LOC105374343	936210	1151506	FGFRL1	1011821	1026898
chr14	LINC00341	95307265	95510090	SYNE3	95416082	95519720
chr2	LOC105373774	183111202	183319628	NUP35	183117489	183161684
chr16	ATP2A1‐AS1	28778487	29025211	CD19	28931734	28939347
chr7	LINC00996	150333653	150548140	GIMAP7	150514856	150521073
chr1	LOC105378604	2969024	3538621	MEGF6	3487941	3624757
chr8	LOC105379219	8001046	8328352	SGK223	8317730	8386444
chr14	LOC105370503	53250170	53950877	BMP4	53949735	53956862
chr11	H19	1847271	2113176	LSP1	1852969	1892263
chr12	LOC105369763	49842673	50046888	FAIM2	49866895	49904275
chr12	LOC105369763	49842673	50046888	RACGAP1	49989161	50033136
chr1	LOC105378726	53126891	53428149	SLC1A7	53087178	53142632
chr1	LOC105378726	53126891	53428149	LRP8	53226891	53328149
chr7	ABHD11‐AS1	73635068	73836000	STX1A	73699204	73719702
chr4	LOC105374528	23617859	23869047	PPARGC1A	23792020	24472975

## 4 DISCUSSION

READ is one of the deadliest malignancies, and the molecular mechanisms underlying the initiation and development of READ remain largely unknown. Hence, comprehensive detailing of its mechanisms is critical. An increasing number of studies have explored the important regulatory effects of lncRNAs on tumor formation and metastasis. Here, DEmRNAs and DElncRNAs in READ were studied using RNA sequencing. A total of 2113 DEmRNAs (809 downregulated and 1304 upregulated mRNAs) and 150 DElncRNAs (81 downregulated and 69 upregulated lncRNAs) between READ and normal tissue were identified. Additionally, we constructed a READ‐specific PPI network, a DElncRNA‐DEmRNA coexpression network and a DElncRNA‐nearby DEmRNA interaction network. In addition, DEmRNAs and DEmRNAs coexpressed with DElncRNAs were functionally annotated.

Coexpression networks have been used in other studies to identify important modules associated with cancer and the functions of the lncRNAs involved within them.^14^ Herein, construction of the DElncRNA‐nearby DEmRNA interaction network showed that the top ten DElncRNAs with the closest DEmRNAs were CCAT1, LOC102723961, LOC105369370, LOC105374879, MIR17HG, UCA1, GAS5, LINC00926, B3GALT5‐AS1, and LINC00482. To our knowledge, besides CCAT1, MIR17HG, UCA1, and GAS5, three upregulated DElncRNAs (LOC102723961, LOC105369370, and LOC105374879) and three downregulated DElncRNAs (LINC00926, B3GALT5‐AS1, and LINC00482) in READ have been reported for the first time, and their biological functions remain unclear.

Most network construction techniques can only address positive correlations in gene expression data, whereas biologically significant genes exhibit both positive and negative correlations.^3^ In this study, positively correlated DEmRNAs and DE1ncRNAs in READ were defined as positively coexpressed DElncRNA‐DEmRNA pairs, and negatively correlated DEmRNAs and DE1ncRNAs were defined as negatively coexpressed DE1ncRNA‐DEmRNA pairs. CDK1, AURKB, CDC6, FOXQ1, NUF2, and TOP2A were the hub proteins of the READ‐specific PPI network. CDK1, a member of the CDKs, is a serine/threonine kinase that promotes the G2‐M transition and regulates G1 progression and G1‐S transition.^15^ CDK1 is overexpressed in human colorectal cancers and relevant to the clinical behavior of human colorectal cancers, which was shown by the association between a high ratio of CDK1 nuclear to cytoplasmic expression and poor overall survival and that CDK1 was an independent risk factor for outcome.^16^, ^17^ AURKB, a member of the aurora kinase family, is an important diagnostic and prognostic marker involved in the carcinogenesis of colorectal cancers.^18^ FOXQ1 is frequently upregulated in colorectal cancers, and FOXQ1 knockdown suppressed cell proliferation and the migration and invasion of colorectal cancers.^19^ TOP2A is a potential predictive biomarker for anthracycline and irinotecan treatment in colorectal cancer, and high frequency of gene gains for the TOP1 and TOP2A genes were reported in colorectal cancers.^20^ Elevated NUF2 expression was associated with poor prognosis in colorectal cancer, and the knockdown of NUF2 expression suppressed the growth of tumor cells.^21^ Therefore, we speculated that CDK1, AURKB, FOXQ1, NUF2, and TOP2A might play important roles in READ. Interaction network analysis showed that AURKB was coexpressed with SNHG5 and that FOXQ1 was coexpressed with LOC105374879. Hence, we further hypothesized that SNHG5 and LOC105374879 might play important roles in READ by regulating AURKB and FOXQ1, respectively.

CCAT1 is upregulated in colorectal cancer but not in normal tissue.^22^ A CCAT1‐specific peptide nucleic acid‐based molecular beacon was reported to serve as a powerful diagnostic tool for the specific identification of colorectal cancer.^23^ GAS5 is associated with not only susceptibility to colorectal cancer but also the metastasis of colorectal cancer to the lymph node.^24^SLCO1B3, a solute carrier organic anion transporter family member, is upregulated in colorectal cancer.^25^ The overexpression of SLCO1B3 changed p53‐dependent pathways and conferred apoptotic resistance in colorectal cancer.^26^ SLCO1B3 protein expression was significantly correlated with proximal tumor location and the expression of mismatch repair genes, and SLCO1B3 was identified as a cell‐surface marker differentially expressed in colon adenocarcinoma relative to its expression in the surrounding normal colon tissue.^27^ In this study, SLCO1B3 was coexpressed with CCAT1 and GAS5. Therefore, we presumed that both CCAT1 and GAS5 might be involved in the development of READ by regulating SLCO1B3.

According to KEGG pathway enrichment analysis of DEmRNAs and DEmRNAs coexpressed with DElncRNAs, the p53 signaling pathway, intestinal immune network for IgA production and colorectal cancer pathway were three READ‐related pathways. MSH6 coexpressed with two DElncRNAs (LOC105374879 and CASC15) and BCL2 coexpressed with B3GALT5‐AS1 were significantly enriched in the colorectal cancer signaling pathway. TNFRSF17 coexpressed with B3GALT5‐AS1 was enriched in the intestinal immune network for IgA production. CCNB2 coexpressed with LOC105374879 was enriched in the p53 signaling pathway. MSH6 is a mismatch repair gene involved in colorectal cancers, and it was reported that most patients with colorectal cancer carrying an MSH6 mutation were diagnosed after the age of 50 and had distally localized tumors. TNFRSF17 may be a candidate gene associated with the pathogenesis of colon cancer, and the haplotypes of TNFRSF17 polymorphisms might be markers for colon cancer susceptibility.^28^ BCL2 is a well‐known protein that prevents apoptosis in many kinds of tumors and is routinely assayed as a diagnostic marker in the clinical practice of pathology. Very recent studies found that BCL2 was downregulated in early‐stage colon adenocarcinoma and that BCL2 was involved in the metastasis of colon adenocarcinoma to the lymph nodes.^29^, ^30^ In our study, BCL2 was reduced in READ, which indicated that BCL2 might regulate READ as well. Therefore, we hypothesized that LOC105374879, CASC15, and B3GALT5‐AS1 might play pivotal roles in READ by regulating the colorectal cancer signaling pathway, the intestinal immune network for IgA production and the p53 signaling pathway.

In summary, we identified 2113 DEmRNAs and 150 DElncRNAs in READ compared to their expression in normal tissues. The PPI network identified several hub proteins including CDK1, AURKB, CDC6, FOXQ1, NUF2, and TOP2A. DElncRNA‐DEmRNA coexpression and DElncRNA‐nearby DEmRNA interaction networks were constructed to identify hub lncRNAs, including CCAT1, LOC105374879, GAS5, and B3GALT5‐AS1. The colorectal cancer pathway, intestinal immune network for IgA production, and p53 signaling pathway were three significantly enriched pathways for DEmRNAs and DEmRNAs coexpressed with DElncRNAs. MSH6 coexpressed with two DElncRNAs (LOC105374879 and CASC15) and BCL2 coexpressed with B3GALT5‐AS1 were significantly enriched in the colorectal cancer signaling pathway of. TNFRSF17 coexpressed with B3GALT5‐AS1 was enriched in the intestinal immune network for IgA production. CCNB2 coexpressed with LOC105374879 was enriched in the p53 signaling pathway. Our results warrant further studies on these mRNAs and lncRNAs to improve our understanding of the mechanisms associated with the pathogenesis and progression of READ. However, there are limitations to our study. First, the sample size for RNA sequencing was small, and large numbers of READ samples are needed for further research. Second, DEmRNAs and DElncRNAs in READ were identified, but their biological functions were not studied. Therefore, in vivo and in vitro experiments are necessary to elucidate the biological roles of DEmRNAs and DElncRNAs in READ in future work.

## 5 CONFLICT OF INTEREST STATEMENT

The authors declare that they have no conflict of interest. No competing financial interests exist.

## DATA AVAILABILITY STATEMENT

The dataset supporting the conclusions of this article is included within the article.
